# AUKF-PINN: An adaptive framework for low-energy gamma multiphase flow measurement under noisy industrial conditions

**DOI:** 10.1371/journal.pone.0355203

**Published:** 2026-08-04

**Authors:** Yibo Huang, Haibo Liang, Liang Zhu, Linhan He, Ting Zeng, Mingyang Liu

**Affiliations:** 1 Southwest Petroleum University, Chengdu, China; 2 China National Petroleum Corporation’s Chuanqing Drilling International Engineering Company, Chengdu, China; 3 China National Petroleum Corporation Tarim Branch Oilfield Oil and Gas Marketing Division, Korla, China; 4 Sichuan Northeast Gas Mine of Southwest Oil and Gas Field Branch of China National Petroleum Corporation, Chengdu, China; Northeastern University, CHINA

## Abstract

Measuring multiphase flow at drilling sites presents numerous challenges, including detector window adhesion, electromagnetic interference, and temperature drift. These factors significantly increase measurement errors when traditional low-energy gamma flowmeters are directly applied in field conditions. To address this problem, this paper proposes an adaptive unscented Kalman filter and physics-informed neural network fusion algorithm (AUKF-PINN) for real-time and accurate measurement of gas-liquid-solid three-phase flow. This algorithm constructs a four-dimensional extended state space model, which includes the linear mass of the gas phase, liquid phase, solid phase, and the thickness of the adhered layer, achieving joint estimation of physical states and interference states; this model precisely handles the exponential nonlinearity of the Beer-Lambert law using unscented Kalman filtering, avoiding linearization errors; and introduces a lightweight physics-informed neural network to learn the dynamic and noise evolution laws of the adhered layer, ensuring that the network output conforms to basic physical laws; furthermore, an adaptive noise fusion strategy based on innovation matching is designed, combining the physical prior of PINN and the Sage-Husa statistical estimation, to ensure that the filter is always in the optimal gain state. Experimental results based on on-site data from the drilling platform show that AUKF-PINN performs extremely well in predicting the fraction ratio of phases and provides an effective physical information-enhanced filtering example for multiphase flow measurement in complex environments.

## 1. Introduction

At the well operation site, the precise measurement of the outlet flow rate has a crucial impact on the accuracy of managed pressure drilling (MPD) [1,2] and the safety identification of overflow risks [3,4] However, the harsh environment at the drilling site and the fact that the outlet flow rate is a multiphase flow with high solid content and high displacement pose a significant challenge to non-separated measurement technology [5,6].

The outlet flow at a drilling site is a strongly transient gas–liquid–solid multiphase flow, whose phase distribution and interfacial structure do not follow a single steady mechanism but are governed by strongly coupled, often non-equilibrium physical processes. Recent studies on petroleum and energy systems have documented this complexity from complementary angles: a fully coupled wellbore–reservoir thermo-hydro-mechanical-diffusion model shows how flow, heat transfer and gas diffusion/dissolution interact dynamically in the wellbore–reservoir system [7], while visual and exergy-based analyses of the in-situ non-equilibrium phase behaviour of CO_2_–oil systems reveal how phase distribution and component partitioning evolve continuously under time-varying pressure and concentration [8]. Such dynamic phase redistribution, non-equilibrium interfacial evolution and strong multiphysics coupling are precisely what make robust field measurement difficult. A measurement method for this application must therefore remain accurate under rapidly changing, non-steady conditions rather than only under steady-state calibration.

In recent years, a variety of sensor technologies have emerged for the measurement of multiphase flows of oil and gas. Common examples include electromagnetic flowmeter measurement technology, mass flow measurement technology, nuclear magnetic work measurement technology, and multi-phase flow measurement technology based on radiation. Non-invasive measurement methods such as electromagnetic flowmeters and ultrasonic flowmeters have high measurement accuracy and can effectively avoid the wear of sensors caused by high flow rates and high solids content of drilling fluids.[9,10] However, since they cannot directly measure mass flow, they have caused problems for subsequent safety measures such as MPD and overflow monitoring. Therefore, these volumetric flowmeters, including turbine flowmeters [11–13], oval Gear flowmeters [14], orifice plate flowmeters [15–17], and vortex flowmeters [18,19], cannot be the optimal choice for measuring the outlet flow of drilling.

The Coriolis mass flowmeter, as the most accurate flowmeter for single-phase flow measurement [20],is a common choice for practical field applications. It is typically used for measuring the outlet flow at the choke manifold. The improvement methods for enhancing the accuracy of Coriolis force multi-phase flow measurement have also become a research hotspot in recent years. The main improvements lie in the structural improvement of the flowmeter and the improvement of data-driven methods. For instance, C. Rolph et al. proposed dividing the mass flowmeter into multiple small-diameter measurement tubes, replacing the original large-diameter measurement tubes by installing them vertically in the direction of fluid flow, and obtaining the mass flow and density of the fluid through the deflection sensor. This method has achieved a significant improvement compared to the previous straight pipe measurement. [21] In terms of data-driven methods, traditional machine learning approaches, such as Support Vector Machines (SVM) [22,23],Random Forest (RF) [24],and Artificial Neural Networks (ANN) [25,26], have achieved good accuracy improvement results. Wasif proposed a Gaussian Regression Process (GPR) driven model to improve the accuracy of solid-liquid two-phase flow measurement. Experimental results show that this method outperforms ANN and SVM for the measurement of solid-liquid two-phase flow [26] However, when the Coriolis force flowmeter is used for measuring gas-liquid two-phase flow, if there is too much gas, it may form slug flow, which causes the vibration tube to fail to vibrate and makes it difficult to fully adapt to complex drilling environments.

In NMR measurement technology, Meribout uses 3-D finite element method (FEM) simulation method to verify the NMR device combined with Halbach array optimized by particle swarm optimization algorithm, and the experimental results show that it has a good match in multiphase flow experiments [27].Meribout proposed an optimal line-echo magnet array design for multiphase flow flowmeters based on NMR and magnetic resonance imaging [28].In radiometry, Song developed a hybrid X-ray density measurement system consisting of a 50 kV, 1 ma double-ray tube and several linear detector arrays for measuring the density of two-phase flows [29]. Bashaher used artificial neural network (ANN) and various feature extraction techniques to improve the accuracy of two-phase flow meters based on double-ray radiation [30], and Taaylan proposed radial basis function neural network to determine the flow pattern and predict the volume fraction of three-phase flow [31].Although these two methods have high accuracy for multiphase flow measurement and are considered to be reliable techniques in multiphase flow measurement, they have not been widely recognized in oil fields because of their potential hazards to the environment and workers.

Low energy gamma flowmeter is a new type of multiphase flowmeter, which is jointly developed by Southwest Petroleum University and Chengdu Yangpai Technology Co., LTD. The total flow rate is measured by differential pressure method, and the phase division ratio is measured based on multi-channel low energy gamma full-section measurement technology. The real-time on-line measurement of oil-gas-water three-phase mixed fluid with no source ray is successfully realized. Under stable multiphase flow conditions, the phase fraction measurement error of the low-energy gamma photons flow meter for oil, gas, water and solid phase substances is within 5.0%. However, in the face of complex noise environment on site, such as electromagnetic noise and detector surface adhesion, it will lead to nonlinear time-varying noise in the measurement of low energy gamma energy intensity, so the energy intensity signal is filtered. Therefore, it is particularly necessary to improve the precision of phase division ratio measurement.

For the signal processing methods of different flow meters, scholars from all circles have made a lot of research to apply to different industrial environments. For example, Chen et al. proposed a method to measure the phase difference of Coriolis flow meters based on extended Kalman filter, which enhanced the anti-interference ability of Coriolis flow meters [32], Jiang et al. proposed a flow estimation method for gas-liquid two-phase flow based on filter-enhanced convolutional neural network (FECNN), which has significant advantages in flow estimation [33]. Shao et al. proposed a digital signal processing method based on piecewise Kalman filtering for vortex flow meters, which can effectively reduce the energy of transient impact interference, thus reducing the difficulty of vortex signal extraction [34]. Li et al. proposed a signal processing method based on peak ratio eigenvalue sequences (PRCVS) for transmitting temporal ultrasonic gas flow meters, which significantly improved the performance of the model in gas flow measurement [35].

Although all the aforementioned filtering algorithms have achieved certain results, they also have certain problems when applied to low-energy gamma flow meters. The Extended Kalman Filter (EKF) approximates nonlinear systems by performing a first-order Taylor expansion on nonlinear functions and conducting local linearization. However, this approximation method is prone to introducing system errors under strong nonlinear absorption conditions, which may cause the estimation to deviate from the true value or even diverge [36–39]. Piecewise Kalman filters (such as the multi-model extended Kalman filter, MMEKF) are designed for impulsive disturbances, and use multiple models to approximately represent the entire state space to reduce large deviations [40]. However, these methods are limited in suppressing the slowly varying baseline drift caused by the adhesion layer. Traditional machine learning methods lack physical constraints to drive, may produce predictions that violate physical laws, and have poor generalization ability to operating conditions outside the distribution of training data [41]. Therefore, this paper proposes an adaptive Unscented Kalman filter-physics-informed neural network (AUKF-PINN) fusion model. The core idea is to use AUKF to realize real-time state estimation in the fast time scale, and PINN to learn the dynamic law of the adhesion layer in the slow time scale, and the adhesion layer thickness is used as the augmented state variable to estimate online, fundamentally separating the environmental interference and the phase holdup signal. The core contributions and innovations of this paper are as follows:

1. Adhesion Layer Augmented State Space and Joint Estimation: By incorporating the thickness of the adhesion layer of the detector window as an explicit state variable into a four-dimensional augmented state space model, the joint online estimation of the linear mass of gas, liquid, and solid phases as well as adhesion interference is achieved. This design fundamentally separates environmental interference from the phase composition signal, solving the baseline drift problem caused by window contamination in traditional methods and achieving real-time compensation for adhesion effects without additional sensors.
2. Dual Physical Constraint Embedded PINN Auxiliary Module: In a lightweight multi-layer perceptron architecture, innovative Beer-Lambert physical residual constraints and mass conservation constraints are introduced to force the adhesion layer thickness output by the network to conform to the laws of photon attenuation and total flow balance. Through the weighted loss function, physical priors and data-driven collaborative training are achieved, enabling the network to learn the noise evolution laws with physical significance from historical data and avoiding the common physical inconsistency problems of pure data-driven methods.
3. Adaptive Noise Fusion Strategy Based on Innovation Matching: A dynamic weight adjustment mechanism based on innovation covariance and the matching degree of PINN’s predicted noise with the Sage-Husa statistical estimation is proposed. The physical prior noise provided by PINN and the Sage-Husa statistical estimation are weighted and fused. This strategy achieves the complementary advantages of predictive physical model and data-driven adaptability: when the adhesion is stable, trust the regular prediction of PINN; when the flow pattern changes suddenly, rely on the real-time feedback of Sage-Husa. This significantly improves the filter’s robustness to non-stationary noise.

**Fig 1:**
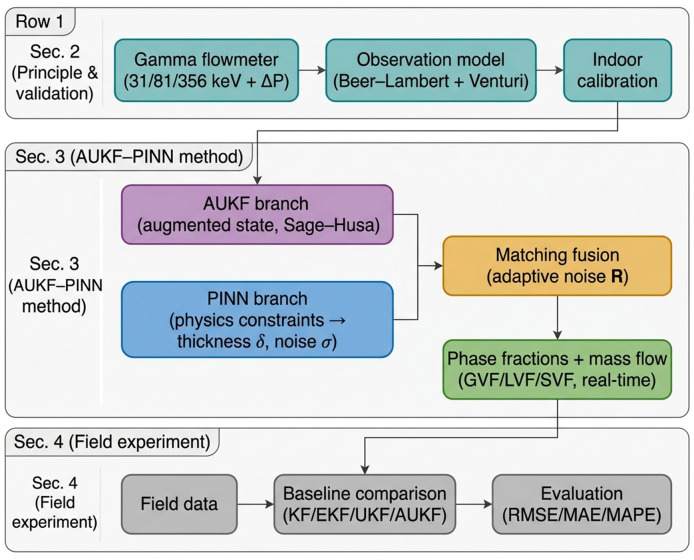
Overall framework diagram.

It is worth clarifying the essential differences between the proposed AUKF-PINN and three related baselines. Compared with the standard UKF, which assumes fixed and known noise statistics, AUKF-PINN augments the state with the adhesion-layer thickness and estimates the observation-noise covariance online, so it can track the slowly varying, state-correlated field noise that a fixed-parameter UKF cannot. Compared with a plain PINN used as a black-box regressor, our PINN does not directly output the phase fractions; instead it supplies a physically-constrained noise prior and adhesion estimate that are fused into a recursive Bayesian filter, thereby retaining the real-time recursion, uncertainty propagation and stability guarantees of Kalman filtering that a stand-alone PINN lacks. Compared with fixed-parameter adaptive filtering, the innovation-matching fusion weight lets the model rely on the physical prior when adhesion is stable and on real-time statistics when the flow pattern changes abruptly, avoiding the gain collapse that purely data-driven adaptation suffers under sudden transients. The incremental contribution is thus the tight, physically-consistent coupling of these three elements rather than any single one in isolation. The overall process of this work is shown in Fig 1.

## 2. Measurement principle and laboratory validation of the low-energy gamma flowmeter

### 2.1. The measurement principle of the low-energy gamma flowmeter

The low-energy gamma flowmeter measures the total mass flow rate Q of multiphase flow by using a Venturi tube, and then measures the phase fraction of the multiphase flow, namely the gas fraction (GMF), water fraction (WMF), and solid fraction (SMF) of the multiphase flow. By multiplying the total flow rate Q and the phase fractions (GMF, WMF, SMF), the mass flow rates of gas, water, and solid can be obtained.

The total mass flow rate Q measured by Venturi is as shown in Equation (1):


$$\begin{array}{c} Q=\frac{C}{\sqrt{\text{1}-\beta^{\text{4}}}}\varepsilon \frac{\pi }{\text{4}}d^{\text{2}}\sqrt{\text{2}\Delta P\rho_{mix}} \end{array}$$ (1)

Where C is the outflow coefficient, with the default value being 0.995; βs the diameter ratio, with the default value being 0.5; εε is the expansion coefficient, with the default value being 1;*d* is the throat diameter, which is a constant value; $\rho_{mix}$ is the density of the mixed liquid;ΔP is the differential pressure of the Venturi tube.

**Fig 2:**
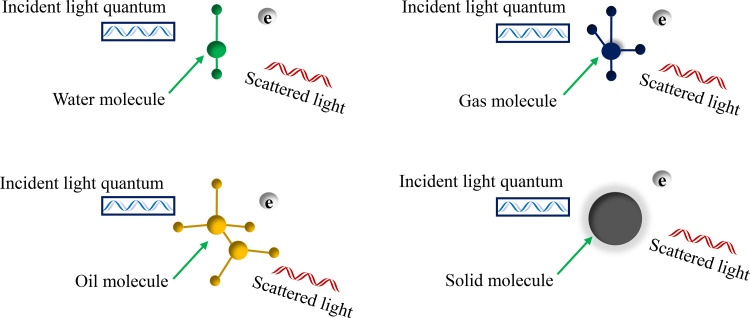
Schematic diagram of the principle of low energy gamma flowmeter.

The core technology of the low-energy gamma flowmeter is the low-energy gamma photons absorption technology. In this absorption technology, the sensor emits a group of low-energy gamma rays (31 kiloelectron volts, 81 kiloelectron volts, and 356 kiloelectron volts) with multiple energy levels. These rays have strong penetrating ability and can penetrate the medium. Due to the absorption effect of the medium, the energy intensity of these rays will weaken. As shown in Fig 2, the low-energy gamma absorption principle of four substances (oil, gas, water, and solid) is demonstrated.

Using low-energy gamma photons scintillation detectors to measure the energy intensity of each group of photons penetrating through the multiphase flow, subsequently, based on the measured energy intensity of the photons, establish the photoelectric cross-section and Compton cross-section equations between the photons and each phase of the substances. Since the main components of the outlet flow at the drilling site are gas, liquid and solid, we have selected three energy levels, namely 31 keV, 81 keV and 356 keV, to establish the measurement method.:

The photoelectric absorption equation for 31 keV optical quanta:


$$\begin{array}{c} \text{ln}\left(\frac{N_{\text{31}}}{N_{x,\text{31}}}\right)=\mu_{g,\text{31}}q_{g,\text{31}}+\mu_{o,\text{31}}q_{o,\text{31}}+\mu_{w,\text{31}}q_{w,\text{31}}+\mu_{s,\text{31}}q_{s,\text{31}}=n_{\text{1}} \end{array}$$ (2)

The photoelectric absorption equation for 81 keV optical quanta:


$$\begin{array}{c} \text{ln}\left(\frac{N_{\text{81}}}{N_{x,\text{81}}}\right)=\mu_{g,\text{81}}q_{g,\text{81}}+\mu_{o,\text{81}}q_{o,\text{81}}+\mu_{w,\text{81}}q_{w,\text{81}}+\mu_{s,\text{81}}q_{s,\text{81}}=n_{\text{2}} \end{array}$$ (3)

The photoelectric absorption equation for 356 keV optical quanta:


$$\begin{array}{c} \text{ln}\left(\frac{N_{\text{356}}}{N_{x,\text{356}}}\right)=\mu_{g,\text{356}}q_{g,\text{356}}+\mu_{o,\text{356}}q_{o,\text{356}}+\mu_{w,\text{356}}q_{w,\text{356}}+\mu_{s,\text{356}}q_{s,\text{356}}=n_{\text{4}} \end{array}$$ (4)

Among them, $N_{\text{31}}$, $N_{\text{81}}$, $N_{\text{356}}$ represent the transmission counts of 31 keV, 81 keV, and 356 keV energy photons in the vacuum pipe without medium, which are the calibration values. $N_{x,\text{31}}$, $N_{x,\text{81}}$, $N_{x,\text{356}}$ represent the transmission counts of 31 keV, 81 keV, and 356 keV energy photons penetrating the gas-liquid-solid multiphase flow medium, which are the measured values. $\mu_{g,\text{31}}$, $\mu_{w,\text{31}}$, $\mu_{s,\text{31}}$ are the linear mass absorption coefficients of gas, liquid, and solid for 31 keV photons. $q_{g,i}$, $q_{w,i}$, $q_{s,i}$((i = 31, 81, 356) are the linear masses of gas, liquid, and solid, where the linear mass refers to the mass of the phase substance that the photon passes through.

By combining formulas (2) – (4), the following results can be obtained:


$$\begin{array}{c} D=\left|\begin{array}{ccc} \mu_{g,\text{31}} & \mu_{w,\text{31}} & \mu_{s,\text{31}}\\ \mu_{g,\text{81}} & \mu_{w,\text{81}} & \mu_{s,\text{81}}\\ \mu_{g,\text{356}} & \mu_{w,\text{356}} & \mu_{s,\text{356}} \end{array}\right|\;{\;D}_{g}=\left|\begin{array}{ccc} n_{\text{1}} & \mu_{w,\text{31}} & \mu_{s,\text{31}}\\ n_{\text{2}} & \mu_{w,\text{81}} & \mu_{s,\text{81}}\\ n_{\text{3}} & \mu_{w,\text{356}} & \;\mu_{s,\text{356}} \end{array}\right| \end{array}$$ (5)

$$\begin{array}{c} D_{w}=\left|\begin{array}{ccc} \mu_{g,\text{31}} & n_{\text{1}} & \mu_{s,\text{31}}\\ \mu_{g,\text{81}} & n_{\text{2}} & \mu_{s,\text{81}}\\ \mu_{g,\text{356}} & n_{\text{3}} & \mu_{s,\text{356}} \end{array}\right|\;{\;D}_{s}=\left|\begin{array}{ccc} \mu_{g,\text{31}} & \mu_{w,\text{31}} & n_{\text{1}}\\ \mu_{g,\text{81}} & \mu_{w,\text{81}} & n_{\text{2}}\\ \mu_{g,\text{356}} & \mu_{w,\text{356}} & n_{\text{4}} \end{array}\right| \end{array}$$

Combining D, $D_{g}$, $D_{o}$, $D_{w}$, $D_{s}$,The linear masses of the three phases – gas, liquid, and solid – are calculated:$q_{g}=\frac{D_{g}}{D}$, $q_{w}=\frac{D_{w}}{D}$, $q_{s}=\frac{D_{s}}{D}$

Furthermore, the mass ratios of gas, liquid and solid can be calculated:


$$\begin{array}{c} GMF=\frac{q_{g}}{q_{g}+q_{w}+q_{s}} \end{array}$$ (6)

$$\begin{array}{c} WMF=\frac{q_{w}}{q_{g}+q_{w}+q_{s}} \end{array}$$ (7)

$$\begin{array}{c} SMF=\frac{q_{s}}{q_{g}+q_{w}+q_{s}} \end{array}$$ (8)

Therefore, the masses of gases, water and solids can be calculated: $Q_{g}=Q\cdot GMF$, $Q_{w}=Q\cdot WMF$, $Q_{s}=Q\cdot SMF$.

In this equation, *Q* represents the total mass flow rate of the multiphase flow, $Q_{g}$ is the mass flow rate of the gaseous phase substances, $Q_{w}$ is the mass flow rate of the aqueous phase substances, and $Q_{s}$ is the mass flow rate of the solid phase substances.

### 2.2. Indoor experiment of low-energy gamma flowmeter

**Fig 3:**
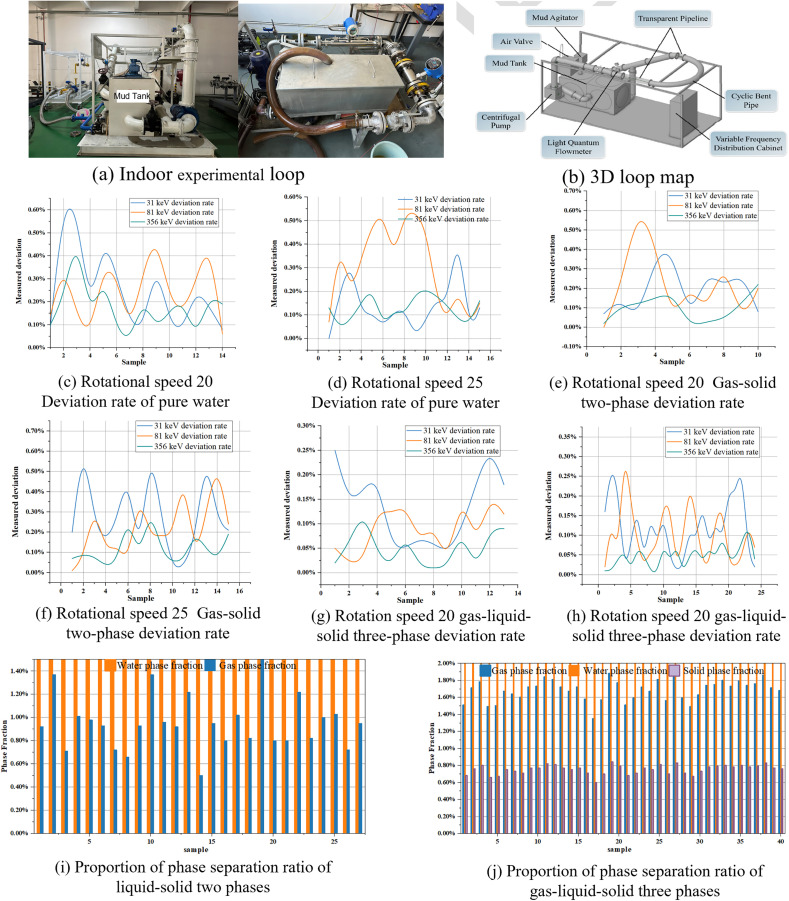
This group of figures shows the indoor experimental setup and test results of the multiphase flow gamma ray measurement system. (a)-(b) show the physical drawing and 3D structure diagram of the experimental bench respectively; (c)-(h) show the deviation rate time series curves of 31 keV, 81 keV and 356 keV energy segments under six working conditions. (i)–(j) present the bar charts of the measured gas-, water- and solid-phase fraction results under the corresponding working conditions. They show that the phase fractions inverted from the three energy segments agree closely with the set reference values across all conditions, with the deviation of each phase kept within the calibration tolerance, which visually confirms the accuracy of the phase-separation inversion under the indoor standard conditions.

We completed the initial equipment verification through an indoor loop. The loop consists of a mud tank, a mud mixer, an air valve, a mud pump, and a low-energy gamma flowmeter. The output frequencies of the mud pump and the mud mixer are controlled by a frequency converter. The output rotation frequency of the mud pump determines the multiphase flow velocity of the loop, while the mud mixer is used to ensure the uniform mixing of the multiphase flow. The physical diagram and 3D model schematic diagram are shown in Fig 3(a).

We set up three sets of working conditions (a, b, c) for the experimental analysis. Each set of conditions included two different flow velocities. Set a was the pure liquid experiment group with a single-direction flow, set b was the gas-liquid two-phase flow experiment group, where the liquid phase accounted for 99% and the gas phase accounted for 1%. Set c was the gas-liquid-solid three-phase flow experiment group, where the liquid phase accounted for 95%, the gas phase accounted for 2%, and the solid phase accounted for 1%.

In the pure water experiment of set a, as shown in Fig 3(c)-Fig 3(d), at the pump rotation speed of 20, the instantaneous water production volume was approximately 16,75 t/h. The average error deviation rates of the three energy segments (31 keV, 81 keV, 356 keV) were 0.27%, 0.23%, and 0.18% respectively. At the speed of 25, the average error deviation rates of each energy segment were 0.14%, 0.30%, and 0.14% respectively. The deviation rates were generally lower than 0.3%, indicating its high performance in the pure liquid experiment.

In the liquid-solid two-phase experiment of set b, the maximum error values of the three energy segments (31 keV, 81 keV, 356 keV) were controlled within 0.66%, and the average values did not exceed 0.28%. Based on the reliable data with low deviation rates (<0.3%) of the three energy segments, the system achieved a high-precision identification of the water phase error <0.05%, proving the accuracy of the indoor calibration. In the three-phase flow C group experiment with the introduction of the gas phase, the deviation rates of the three energy segments were further reduced, with the maximum value being only 0.33% and the average value dropping to the range of 0.04%−0.13%. This indicates that the system can still maintain high-precision measurement when dealing with multi-phase mixed media.

This indoor experiment fully verified the excellent performance of the gamma ray measurement system based on the three energy segments (31 keV/81 keV/356 keV). The deviation rates of the three energy segments were all controlled within 0.7%, indicating that the system has extremely high measurement accuracy, stability, and repeatability under the indoor standard working conditions. It has good robustness to the composition and flow type changes of the medium. Relying on the reliable data from the three-energy segment collaborative measurement, the system achieved high-precision phase separation rate identification with water phase error <0.6% and solid phase error <0.3%, fully meeting the technical requirements of multi-phase flow industrial measurement.

## 3. AUKF-PINN methodology

The laboratory experiments in Chapter 2 show that the measurement accuracy of the low energy gamma flowmeter for gas-liquid-solid three-phase flow in a controlled environment can reach less than 0.5%, which verifies the feasibility of the principle. However, the harsh environment of the drilling site, there are complex noise sources such as detector window adhesion, electromagnetic interference, and temperature drift, which lead to measurement errors when directly applying the indoor model. Therefore, it is crucial to design a filtering algorithm that can adapt to the field noise environment and compensate the interference in real time.Compared with indoor environment, on-site noise has two significant characteristics: non-stationarity and physical correlation. The non-stationarity is reflected by the change of noise statistical characteristics with time. For example, the thickening of adhesive layer leads to the gradual increase of noise variance in low-energy channels. The physical correlation shows that there is a coupling relationship between noise and process states (such as phase holdout and temperature), for example, high solid content will aggravate window adhesion. Traditional fixed-parameter filtering methods (such as the standard Kalman filter) or purely data-driven machine learning models are difficult to cope with these two challenges at the same time.

In order to solve the above problems, this chapter proposes a fusion algorithm of adaptive Unscented Kalman filter (AUKF) and physics-informed neural network (PINN). The core idea of the algorithm is as follows: using AUKF to realize real-time state estimation and tracking, and handling the nonlinearity of the observation equation; PINN is used to learn the physical laws of adhesion layer dynamics and noise statistics to provide adaptive parameters for AUKF. The combination of the two methods significantly improves the robustness of the system to field non-stationary noise while ensuring the real-time performance.

### 3.1. Unscented Kalman filter

The Kalman filter recursively estimates the internal state of the system through the state space model in the observation containing noise. For linear Gaussian systems, it is the optimal estimator. However, the observation equation of the low energy gamma flowmeter (Equations 2−4) is a strongly nonlinear function in exponential form. The Extended Kalman filter (EKF) locally linearizes the system by performing a first-order Taylor expansion at the current estimation point. However, this method has two main drawbacks: 1) in the high attenuation region with large total absorption coefficient, the curvature of the nonlinear function is large, and the linear approximation error is significant; 2) The complex Jacobian matrix needs to be calculated, which increases the difficulty of implementation and the risk of errors when the model is complex

The Unscented Kalman filter (UKF) provides a more elegant way to deal with nonlinearities. The key idea is to use a deterministic sampling point to capture the probability distribution of the mean and covariance states, propagate these points directly through the true nonlinear function, and then recalculate the mean and covariance based on the propagated points. This method can approximate the statistical properties of any nonlinear transformation with second-order accuracy without computing the Jacobian matrix [42].

For the n-dimensional state vector, the UT transformation selects 2n+1 Sigma points, whose mean is $\overset{―}{𝐱}$, and covariance is ${𝐏}_{xx}$. The specific sampling points $\chi_{i}$ of the state equation can be expressed as:


$$\begin{array}{c} \chi_{\text{0}}=\bar{𝐱} \end{array}$$

$$\begin{array}{c} \chi_{\text{i}}=\bar{𝐱}+(\sqrt{(\text{n}+\lambda ){𝐏}_{𝐱𝐱}}{)}_{\text{i}},\text{ i}=\text{1},\ldots ,\text{n} \end{array}$$ (9)

$$\begin{array}{c} \chi_{\text{i}}=\bar{𝐱}-(\sqrt{(\text{n}+\lambda ){𝐏}_{𝐱𝐱}}{)}_{\text{i}},\text{ i}=\text{n}+\text{1},\ldots ,\text{2n} \end{array}$$

$\lambda =\alpha^{\text{2}}(n+\kappa )-n$, s a scaling parameter, which determines the distribution range of the Sigma points around the mean. κ is the secondary scaling parameter. Each point has a corresponding weight, which is used to calculate the weighted mean and covariance.

For the measurement of light quantum multiphase flow, the state (gas, liquid, and solid phase mass) is related to the observation (photon count) through the Beer-Lambert law, which is a nonlinear function. The UKF directly and precisely handles this nonlinearity through the UT transformation, avoiding the linearization error of the EKF. At the same time, its sampling mechanism can better handle the propagation problem of non-Gaussian noise, which is particularly beneficial for the Poisson-Gaussian mixed noise characteristics of count values. Therefore, choosing the UKF as the baseline model of this filtering framework is accurate and efficient.

However, although the standard UKF can effectively handle nonlinearity, its performance depends on accurate noise statistics. Therefore, the time-varying nature of the on-site noise requires to be adaptive. To this end, we constructed an augmented model that includes physical states and disturbance states, and its state vector can be expressed as::


$$\begin{array}{c} X\left(k\right)=[q_{g},q_{l},q_{s},\delta attach{]}^{T} \end{array}$$ (10)

Here, $q_{g},q_{l},q_{s}$ represents the linear mass of the gas, liquid and solid phases, δ attach represents the equivalent thickness of the window adhesion layer, and the observation vector can be expressed as:


$$\begin{array}{c} Z(k\text{)}=[N_{31},N_{81},N_{356},\Delta P{]}^{T} \end{array}$$ (11)

In the formula, $N_{31},N_{81},N_{356}$ represents the actual photon transmission count of the 31 keV, 81 keV, and 356 keV energy channels, and ΔP represents the pressure difference measured by the low-energy gamma flowmeter. The significance of this setting lies in ensuring that the observation dimension is equal to the state dimension, ensuring complete observability in the actual measurement, achieving the deterministic solution of the gas-liquid-solid three-phase system. Considering that the flow rate at the drilling outlet changes relatively smoothly and there is a dynamic balance of accumulation and shedding of the adherent layer, a random walk model with attenuation factors is adopted. Specifically, it can be expressed as:


$$\begin{array}{c} X\text{(k)}=𝐅\left(X\left(k-1\right)\right)+𝐰\left(K\right) \end{array}$$ (12)

Here,W(k) represents the process noise, 𝐅=diag(1,1,1,γ), for $q_{g},q_{l},q_{s}$, he diagonal elements of the state transition matrix are set to 1, indicating that within adjacent sampling intervals, its changes are mainly driven by the observation updates, and the process noise term is used to describe the uncertainty under this assumption. The introduction of the attenuation factor γ reflects the dynamics of the adhesive layer, with a value of 0.99, meaning that the model prediction value will be slightly lower than the previous moment. This can prevent the adhesive layer state from growing indefinitely in the absence of observation updates, enhancing the numerical stability. After the observation state vector and observation vector are constructed, the physical connection between the two vectors is established through the Beer-Lambert law. Specifically, it can be expressed as:


$$\begin{array}{c} h_{i}\left(𝐱\left(k\right)\right)=N_{0,}iexp(-\mu_{g,i}q_{g}-\mu_{l,i}q_{l}-\mu_{s,i}q_{s}-\mu_{att,i}\delta_{attach}),\text{\textbackslash{}hspace\{1em}\}i=1,2,3 \end{array}$$ (13)

$N_{0,i}$ represents the baseline projection count under the condition of air traffic control, while, $\mu_{g,i},\mu_{l,i},\mu_{s,i}$ is the mass absorption coefficient for the gas phase, liquid phase, and solid phase for the i-th energy photon, and for the i-th type of energy photon, $\mu_{att,i}$, is the mass absorption coefficient of the drilling fluid. For the observed vector differential pressure, it is obtained based on the Venturi tube differential pressure principle and through the combined reasoning of the three flow mass flow rate and density, as follows:


$$\begin{array}{c} h_{\text{4}}\left(𝐱\left(k\right)\right)=K\cdot \sqrt{\rho_{mix}\left(𝐱\left(k\right)\right)} \end{array}$$ (14)

$$\begin{array}{c} \rho_{mix}=(q_{g}+q_{l}+q_{s})/(q_{g}/\rho_{g}+q_{l}/\rho_{l}+q_{s}/\rho_{s}) \end{array}$$ (15)

$$\begin{array}{c} K=\frac{C}{\sqrt{1-\beta^{\text{4}}}}\;\cdot \frac{\pi d^{\text{2}}\;}{\text{4}}\;\cdot \sqrt{2\Delta P}\; \end{array}$$ (16)

The geometric constants of the venturi tube, the outflow coefficient, etc. are merged into a constant k, and, $\rho_{g},\rho_{l},\rho_{s}$ is the true density of the gas phase, liquid phase, and solid phase under operating conditions, respectively. After completing the UKF update step, we introduce the Sage-Husa adaptive estimation algorithm into the UKF framework to form the Adaptive UKF (AUKF). The recursive form of the Sage-Husa estimator is as follows.


$$\begin{array}{c} {\hat{R}}_{k}^{SH}=(1-d_{k}){\hat{R}}_{k-1}^{SH}+d_{k}(e_{k}e_{k}^{T}-P_{ZZ,k|k-1}) \end{array}$$ (17)

$$\begin{array}{c} d_{k}=(1-b)/(1-b^{k+1}) \end{array}$$ (18)

$$\begin{array}{c} {𝐞}_{k}=Z\left(k\right)-\hat{h(}k|k-1) \end{array}$$ (19)

n the formula, ${𝐑}_{k}$ s the covariance of the observation noise, $d_{k}$ is the fading forgetting factor, usually ranging from 0.95 to 0.99, to make the estimator focus more on recent data and thus track the changes in noise. $e_{k}$ is the information containing the observation noise. $P_{ZZ,k|k-1}$ s the observation covariance predicted by the UKF.

### 3.2. Adaptive unscented Kalman filter

In the AUKF model constructed in the previous section, its Sage-Husa estimator is completely data-driven. When the system state changes abruptly or the model has short-term errors, the noise covariance may be overestimated, and then the filter gain is too small and the tracking ability is lost. In order to overcome the vulnerability of pure data-driven adaptive methods and exploit the correlation between noise and physical states, a physics-informed neural network (PINN) module was introduced based on the AUKF model. It aims to provide prior information with robustness and physical meaning.

The physics-informed neural network (PINN) proposed by Raissi et al. is a computational method that combines physical constraint equations with neural networks [41], and has a powerful ability to learn implicit patterns from sequence data [43–45]. By embedding the physical laws as constraints into the network training, it can ensure that the learned patterns are consistent with the basic laws of energy decay and mass conservation, and avoid generating predictions that violate the physical laws. We design a lightweight PINN model to enable low-latency forward inference.

In terms of input features, we selected a selection of an observation sequence ${ℐ}_{k}$ containing the current and the past L moments as a time window, and the network structure used a multi-layer perceptron (MLP) containing two hidden layers. The activation function uses ReLU. The number of neurons in the input layer corresponds to the dimension of ${ℐ}_{k}$.

The output side includes the estimated thickness of the adhesion layer $\delta_{attach}^{NN}$ and the standard deviation of the observation noise of each channel $\left[\sigma^{NN}31,\sigma^{NN}81,\sigma^{NN}356,\sigma^{NN}\Delta P\right]$, which constitutes the diagonal noise covariance matrix $𝐑\text{(}k\text{)}=\text{diag}((\sigma^{NN}i{)}^{\text{2}})$. The core of PINN is the physical constraint embedded in the loss function, and then guides the network to learn features that conform to physical laws. This paper designs two physical constraints, corresponding to low-energy gamma photons attenuation and mass conservation respectively

The contribution of the Beer-Lambert physical residual constraint is to enforce that the adhesive layer thickness of the network output is self-consistent with the AUKF current state estimate at the Beer-Lambert equation level. The residual for the ith energy channel is defined as:


$$\begin{array}{c} R_{BL,i}=\text{ln}\left(\frac{N_{\text{0},i}}{N_{i,k}}\right)-\sum_{j=g,l,s}\mu_{j,i}{\hat{q}}_{j,k}-\mu_{att,i}{\hat{\delta }}_{attach},\;i\in \{\text{3}\text{1},\text{8}\text{1},\text{3}\text{5}\text{6}\} \end{array}$$ (20)

$$\begin{array}{c} L_{BL}=\frac{\text{1}}{\text{3}M}\sum_{k=\text{1}}^{M}\sum_{i}\frac{R_{BL,i}^{\text{2}}}{{\left(\sigma_{i,k}^{NN}\right)}^{\text{2}}} \end{array}$$ (21)

The denominator uses the noise variance predicted by the network for weighting, achieving adaptive physical constraints. In high-noise channels (such as at 31 keV during severe adhesion), a larger tolerance for residuals is allowed. This constraint establishes a physical coupling between the network output and the AUKF state estimation, ensuring that the adhesion layer estimation does not violate the basic laws of photon attenuation.

The contribution of the law of conservation of mass lies in that, through the Venturi equation, the thickness of the adhered layer output by the network is indirectly correlated with the measured total flow value. The mixed density is calculated using the AUKF state estimation ${\hat{q}}_{j,k}$ The mass conservation constraint $L_{mass}$ can be expressed as:


$$\begin{array}{c} L_{mass}=\frac{\text{1}}{M}\sum_{k=\text{1}}^{M}{\left(Q_{total,k}-\text{K}\sum_{j=g,l,s}{\hat{q}}_{j,k}\cdot v_{mix}\right)}^{\text{2}} \end{array}$$ (22)

$v_{mix}$ represents the flow velocity, which can be measured using a Venturi tube. This constraint indirectly forces the thickness of the adhered layer output by the network to be consistent with the total flow calculated using the Venturi equation, reflecting the fundamental physical principle of mass conservation. Therefore, its total loss function is:


$$\begin{array}{c} L_{total}=L_{data}+\lambda_{BL}L_{BL}+\lambda_{mass}L_{mass} \end{array}$$ (23)

$L_{data}$ is the data fitting loss, $\lambda_{BL}$ and $\lambda_{mass}$ are the hyperparameters of two physical constraints in the model. The optimizer uses Adam, with the initial learning rate set to 0.01 and the batch size set to 64.

Physical justification of the two constraints. The two constraints are not merely loss terms; each encodes a mechanism specific to low-energy gamma multiphase-flow measurement. The Beer–Lambert residual constraint follows from the exponential photon-attenuation law: a contamination/adhesion layer of equivalent thickness on the detector window adds an extra, energy-dependent attenuation term μ_att,i·δ_attach to every channel, and this term is strongest in the low-energy (31 keV) channel because the mass-attenuation coefficient of the barite-bearing adhesion layer rises sharply as photon energy decreases. Enforcing the residual therefore forces the network to attribute the slowly-varying baseline drift to the adhesion state δ_attach rather than to spurious changes in phase holdup, which is exactly the physical coupling that separates interference from signal. The mass-conservation constraint links the phase linear-masses estimated from the gamma channels to the independent total mass flow obtained from the Venturi differential pressure; because the two measurements share no common noise source, requiring their consistency suppresses non-physical solutions in which the network compensates an attenuation error by an unbalanced phase split. In short, the constraints adapt to gamma multiphase flow because they embed (i) the energy-selective attenuation of the adhesion layer and (ii) the cross-sensor mass balance, both of which are intrinsic to this measurement principle rather than generic regularisers.

### 3.3. AUKF-PINN

he integration of PINN and AUKF is crucial, aiming to enable the physical constraints of PINN to assist the statistical decision-making of AUKF. The core lies in weighting and fusing the noise covariance ${𝐑}_{k}^{NN}$ predicted by PINN with the ${\hat{𝐑}}_{k}^{SH}$ output by the Sage−Husa estimator in AUKF, and using it as the final observed noise covariance ${𝐑}_{k}^{fuse}$, fuse for the algorithm update steps. The specific expression is as follows:


$$\begin{array}{c} {𝐑}_{k}^{fuse}=\alpha_{k}{𝐑}_{k}^{NN}+\left(\text{1}-\alpha_{k}\right){\hat{𝐑}}_{k}^{SH} \end{array}$$ (24)

The fusion weight $\alpha_{k}$ adaptively adjusted according to how well the innovation covariance matches the PINN prediction noise. The theoretical value of innovation covariance is defined as ${𝐏}_{zz,k|k-1}$, When the real-time sample estimate ${𝐞}_{k}{𝐞}_{k}^{T}$ is close to ${𝐑}_{k}^{NN}$ it indicates that PINN prediction is accurate, and its weight should be increased, otherwise α_k should be reduced. Therefore, the following adaptive law is adopted in this paper:


$$\begin{array}{c} \alpha_{k}=\text{exp}(-\beta \cdot \left\|{𝐞}_{k}{𝐞}_{k}^{T}\;{𝐑}_{k}^{NN}\right\|F) \end{array}$$ (25)

**Fig 4:**
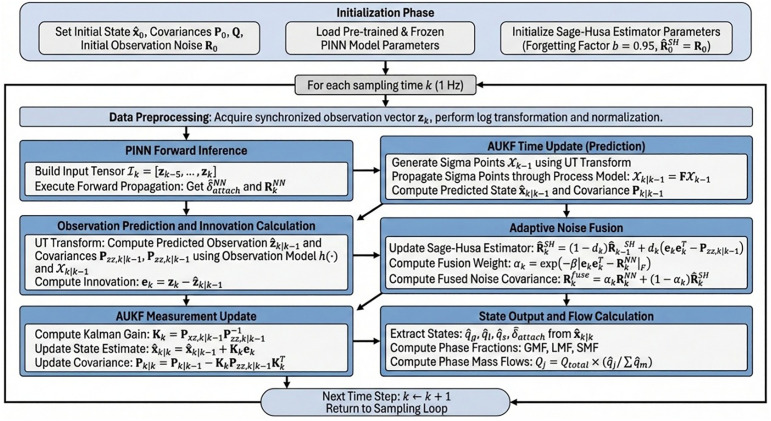
This model is based on the unsupervised Kalman filtering framework and incorporates the Sage-Husa adaptive algorithm. It designs a lightweight PINN auxiliary module. By integrating the Beer-Lambert physical residuals with the mass conservation constraints and the adaptive noise fusion strategy, it combines the physical prediction of PINN with the statistical estimation of Sage-Husa, forming an enhanced filtering framework with AUKF as the recursive core and PINN as the feedforward auxiliary.

Here $|\cdot {|}_{F}$ represents the Frobenius norm, −β s the sensitivity parameter. This function satisfies that when ${𝐞}_{k}{𝐞}_{k}^{T}$ is close to ${𝐑}_{k}^{NN}$, $\alpha_{k}$ approaches 1,and conversely, $\alpha_{k}$ approaches 0,At this point, the filtering model framework is completed. The specific flowchart is shown in Fig 4. This strategy achieves the complementary advantages of physical prior and data-driven. The noise evolution law learned by PINN provides the expected noise level, and Sage-Husa provides real-time feedback on the measured noise based on the real-time innovation. The fusion of the two enables AUKF to quickly adapt to slowly varying noise and increase the robustness of the model.

## 4. Model experiment

In order to verify the effectiveness of the AUKF-PINN fusion filtering algorithm proposed in Chapter 3 in the field measurement of gas-liquid-solid three-phase flow, this chapter conducts experimental verification based on the real operation data collected from a drilling platform in Sichuan. Firstly, the data acquisition scheme and the method for obtaining reference true values are introduced; secondly, the application effect of AUKF-PINN is demonstrated through the calculation of phase fraction and the output display of mass flow rate; finally, through multi-index comparison with extended Kalman filter (EKF), standard unscented Kalman filter (UKF), and adaptive unscented Kalman filter without PINN assistance (AUKF), the accuracy advantages and engineering practicability of this algorithm are comprehensively evaluated.

### 4.1. Data collection

**Fig 5:**
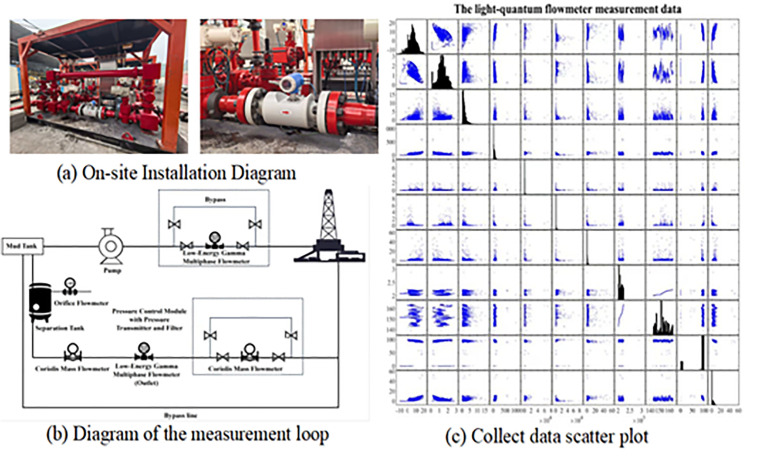
On-site experimental equipment and data scatter plot.

The on-site experiment was conducted in August 2024 on a drilling platform in Sichuan. A DN100 low-energy gamma flowmeter was installed in the downstream outlet straight section of the choke manifold, approximately 3 meters away from the choke valve, to measure the outlet flow rate of the drilling fluid. The on-site installation diagram is shown in Fig 5(a). The fluid was a three-phase mixture of gas, liquid, and solid after throttling and pressure reduction. The collection time was at a well depth of approximately 6000 meters, with a drilling fluid density of 1.2. Due to geological factors, it contained associated gas, so the three-phase mixture of gas, liquid, and solid had associated gas as the gas phase, water-based drilling fluid as the liquid phase, and the drilling fluid contained barite and some high-molecular polymers as the solid phase. The on-site loop consisted of a mud pump, a mud tank, a low-energy gamma flowmeter, a separation tank, and a high-precision Coriolis mass flowmeter. In the experimental loop, the gas-liquid mixture passed through the photon multi-phase flowmeter and then entered the separation tank, where gas-liquid separation was achieved through gravity sedimentation. The liquid phase was measured by the first Coriolis force mass flowmeter, and the gas phase was measured by the orifice plate flowmeter. The two were combined as the standard data. The specific loop diagram is shown in Fig 5(b).

The on-site collected data included time, volumetric gas content, instantaneous gas flow rate, instantaneous liquid flow rate, instantaneous solid flow rate, absolute pressure, differential pressure, medium temperature, 31 keV count, 81 keV count, 356 keV count, total count, volumetric solid content, volumetric liquid content, mixed density. We represent these data using a data scatter plot, as shown in Fig 5(c):

For reproducibility, the key parameters of the field data set are summarised as follows: the total number of collected samples was 1014, the low-energy gamma flowmeter recorded at a fixed sampling interval of ≈120s (about 2 min, i.e., a sampling rate of ≈0.0082 Hz), and the net data-acquisition duration was ≈ 34 h.

### 4.2. Model application effect

To systematically evaluate the comprehensive performance of the AUKF-PINN fusion filtering algorithm in actual drilling conditions, this section conducts verification analysis based on the field data collected. The complexity of the field data lies in multiple aspects such as the interweaving of multi-source noise, the time-varying accumulation of adhering layers, and the intense fluctuations of flow patterns. Therefore, it is difficult to quantify the algorithm’s superiority or inferiority solely through visual observation. Statistical indicators must be introduced to compare the filter output with the reference true value (obtained by combining the standard values after passing through the Coriolis force mass flowmeter and orifice plate flowmeter). We conduct the analysis from two dimensions: signal smoothing degree and phase separation prediction accuracy. The core evaluation criteria are root mean square error (RMSE), mean absolute error (MAE), and accuracy based on relative thresholds. These indicators can depict the fidelity and robustness of the filtering algorithm from different perspectives, thereby comprehensively demonstrating the applicability and engineering value of AUKF-PINN in multiphase flow measurement..

In quantitative evaluation, RMSE is highly sensitive to extremely large errors and can effectively reflect the deviation degree of the algorithm under the worst conditions. It is particularly suitable for scenarios that require strict monitoring of early overflow warnings and other situations with sudden deviations. By measuring the average deviation amplitude, it provides a more intuitive scale for errors and is less affected by individual outliers, making it suitable for evaluating the overall prediction level. In addition, for the absolute error of the oilfield phase content rate not exceeding 3%, we designed an accuracy evaluation index - total prediction accuracy (TPA). For each predicted value with an absolute error less than 3%, it is considered as a correct prediction. TPA is the number of correct predictions divided by the total number, and its specific expression is as follows:


$$\begin{array}{c} Accuracy=\frac{num(pred=true)}{total} \end{array}$$ (26)

**Fig 6:**
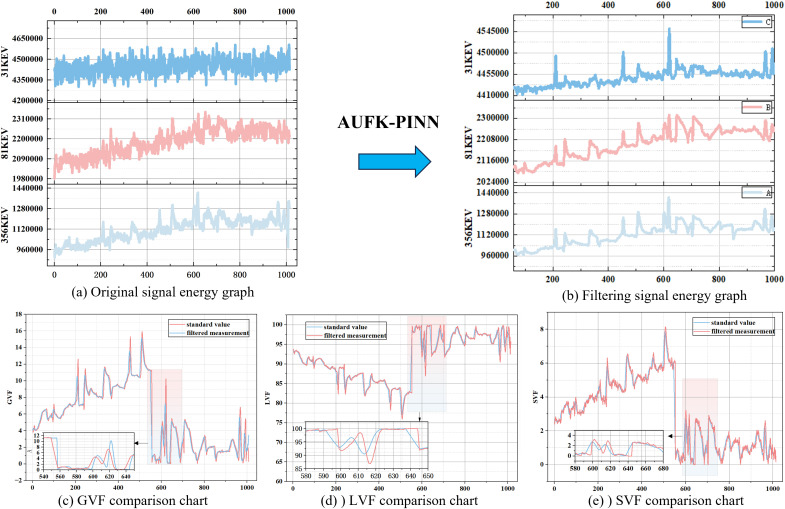
Performance verification of the AUKF-PINN algorithm in the low-energy gamma multi-phase flowmeter. (a) The original photon count signals at three energy levels (31 kiloelectron volts, 81 kiloelectron volts, and 356 kiloelectron volts), showing significant random fluctuations and baseline drift due to window contamination and environmental noise; (b) The filtered signal after AUKF-PINN processing, demonstrating effective noise suppression and stable baseline, while retaining the dynamic flow characteristics; (c) (e) Comparison of the predicted phase fractions (GVF, LVF, and SVF) with the standard values of the reference measurement based on the separator.

The experimental results demonstrate that the AUKF-PINN model exhibits outstanding filtering performance in processing low-energy gamma photons energy level signals. By comparing Fig 6(a) and 6(b), it is clearly observable that the original 31 keV, 81 keV, and 356 keV energy level count signals all show significant fluctuations and drifts. Among them, the 31 keV low-energy channel is most severely affected by the adhering layer, with the count rate showing a slow downward trend accompanied by high-frequency noise. After being processed by the AUKF-PINN algorithm, the spikes of the three energy level signals are effectively suppressed, the baseline becomes stable, and the key dynamic characteristics of the gas-liquid-solid three-phase change are retained. This filtering effect is attributed to the collaborative effect of the dual architecture: the unscented Kalman filter precisely propagates the exponential nonlinearity of the Beer-Lambert equation through Sigma points, avoiding the first-order approximation error of the extended Kalman filter; the physics-informed neural network adaptively learns the accumulation law of the adhering layer and dynamically adjusts the observation noise covariance, keeping the filter always in the optimal gain state. Particularly, the algorithm effectively filters out environmental noise without causing significant phase lag in the actual flow pattern changes, ensuring the real-time performance of dynamic tracking and laying a reliable signal foundation for subsequent phase fraction inversion..

In terms of the measurement accuracy of phase fraction, the AUKF-PINN model also performs exceptionally well. The solid phase fraction prediction achieves 98.01% accuracy with an RMSE of only 0.47, reflecting the strong attenuation characteristics of high-density solids in the 356 keV Compton scattering regime; the prediction accuracy of the volume gas fraction and volume liquid fraction reaches 93.55% and 90.07% respectively, with an RMSE within 1.03% and 1.51% respectively, and the three-phase prediction errors are far superior to the accuracy threshold of the fine control pressure drilling field application. Particularly noteworthy is that under the severe fluctuation of the gas phase in the 600–800 data segment, the model effectively suppresses the baseline drift caused by window contamination through online estimation and compensation of the adhering layer state, making the filtering output closely follow the changes in the standard value, without the common cumulative error divergence phenomenon of traditional methods. Compared to the inherent defect of the Coriolis force flowmeter that it becomes unstable due to the vibration tube gas-particle effect when the gas fraction exceeds 7%, this model maintains continuous and stable measurement within the GVF 0–90% range and does not require interruption of operations for detector cleaning or calibration. In summary, the AUKF-PINN algorithm combines high precision, strong robustness, and real-time performance, fully meeting the engineering application requirements of drilling outlet flow monitoring, and can provide reliable data support for early overflow warning, control pressure parameter optimization, and wellbore safety decision-making.

### 4.3. Model comparison

This section will further verify the performance advantages of the AUKF-PINN fusion filtering algorithm compared to traditional filtering methods. In this section, the Kalman filter (KF), Extended Kalman filter (EKF), Unscented Kalman filter (UKF), and the adaptive Unscented Kalman filter without PINN assistance (AUKF) are selected as the comparison benchmark models.

To comprehensively evaluate the performance of each filtering algorithm in the measurement of gas-liquid-solid three-phase flow, this section conducts a horizontal comparison of the five models – AUKF-PINN, AUKF, UKF, EKF, and KF – from two dimensions: error amplitude and prediction accuracy. Root Mean Square Error (RMSE), Mean Absolute Error (MAE), Mean Absolute Percentage Error (MAPE), and accuracy based on 2% and 3% absolute deviation thresholds are used as evaluation indicators. RMSE is sensitive to outliers and reflects the deviation of the algorithm under the worst conditions; MAE measures the average deviation amplitude, which is closer to the engineering intuition; MAPE represents the error proportion in a relative form, facilitating cross-comparison; accuracy directly depicts the proportion of samples that meet the industrial error tolerance, and is a key indicator of engineering practicability. Table 1 summarizes all the comparison results of the models on the three phases (gas phase, liquid phase, solid phase).

**Table 1:** Indicator model comparison table.

		AUKF-PINN	AUKF	UKF	EKF	KF
RMSE	GVF	1.03	1.88	2.38	2.19	3.01
	LVF	1.51	2.01	2.83	3.05	3.94
	SVF	0.46	1.17	1.76	1.85	2.09
MAE	GVF	0.50	0.89	1.59	1.68	2.46
	LVF	0.76	1.02	2.33	1.97	2.85
	SVF	0.25	0.73	0.88	0.79	1.38
MAPE	GVF	0.195	0.362	0.572	0.563	0.690
	LVF	0.300	0.451	0.670	0.722	0.788
	SVF	0.147	0.235	0.583	0.565	0.635
Accuracy rate (2%)	GVF	93.55	90.65	87.14	85.15	80.57
	LVF	90.10	89.18	86.28	83.08	81.4
	SVF	98.01	93.58	90.22	86.25	81.31
Accuracy rate (3%)	GVF	96.92	92.19	90.12	89.64	86.55
	LVF	94.34	90.33	89.03	87.25	87.10
	SVF	98.70	96.12	94.23	91.78	88.20

Table 1 clearly shows that AUKF-PINN significantly outperforms the comparison models in all indicators. In terms of error amplitude, the gas phase RMSE of AUKF-PINN is 1.03, which is 45.2% lower than that of the less optimal AUKF (1.88); the liquid phase RMSE is 1.51, which is 24.9% lower than that of AUKF (2.01); the solid phase RMSE is only 0.46, which is 60.7% lower than that of AUKF (1.17). MAE and MAPE also show similar advantages, especially the solid phase MAPE is as low as 0.147, indicating that the model’s prediction of solid phase content is almost unbiased. In terms of the most concerned accuracy in engineering, AUKF-PINN achieves an accuracy of 93.55% for the gas phase at the 2% threshold, 90.10% for the liquid phase, and 98.01% for the solid phase; at the 3% threshold, the solid phase accuracy further increases to 98.70%, and the gas phase and liquid phase also reach 96.92% and 94.34%, respectively. In contrast, the traditional KF’s accuracy at the 2% threshold is generally lower than 87%, while EKF and UKF have certain improvements, but still have a gap compared to AUKF-PINN. This indicates that AUKF-PINN effectively suppresses multi-source noise interference by introducing the physics-informed neural network for online learning of the dynamic laws of the adhesion layer, significantly improving the estimation accuracy and stability of multi-phase flow parameters and fully meeting the strict requirements of the drilling site for outlet flow monitoring.

**Fig 7:**
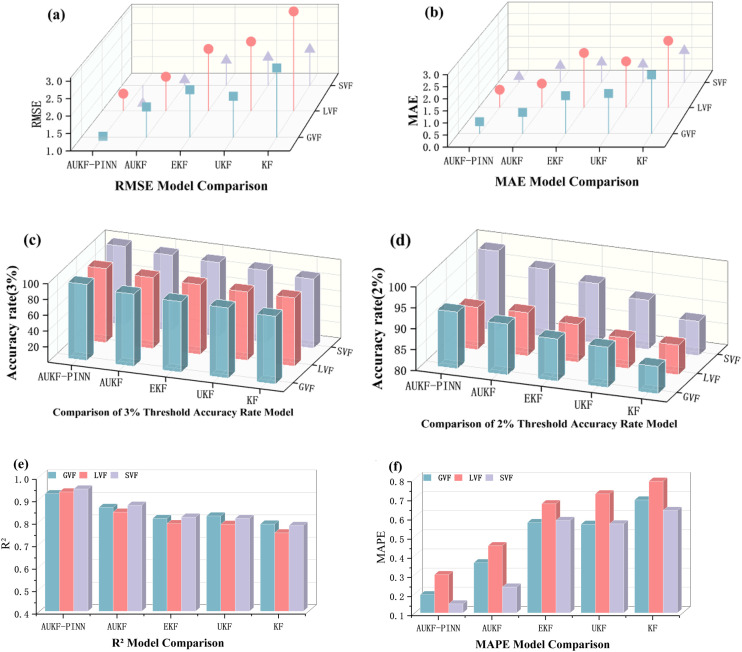
Model Performance Comparison Chart.

Fig 7 presents the distribution of indicators for five filtering algorithms in the gas phase, liquid phase, and solid phase in a three-dimensional graph. From the figure, it is clearly observable that the indicators corresponding to AUKF-PINN are optimal in all three phases, indicating that AUKF-PINN has strong anti-interference ability and high-precision estimation advantages in complex multiphase flow environments, providing an intuitive basis for subsequent engineering applications.

## 5. Conclusion

This paper addresses the problem of reduced measurement accuracy of on-site low-energy gamma multiphase flow meters caused by multi-source, time-varying industrial noise, and proposes an adaptive unscented Kalman filter fused with a physics-informed neural network (AUKF-PINN). The algorithm constructs a four-dimensional augmented state of the gas, liquid, solid and adhesion-layer variables, exploits the AUKF for high-precision nonlinear propagation of the Beer–Lambert observation model, embeds physical residual constraints in the PINN to learn the noise characteristics, and adaptively fuses the two noise estimates to keep the filter at its optimal gain in real time. The main conclusions, grouped by their respective effects, are summarized as follows.

(1) Three-phase fraction measurement accuracy. For the solid phase, the RMSE is only 0.46, with accuracies of 98.01% and 98.70% at the 2% and 3% thresholds, respectively; for the gas and liquid phases, the accuracies reach 96.92% and 94.34%, while the RMSE is controlled within 1.03% and 1.51%. The method therefore attains high and well-balanced accuracy across all three phases, fully meeting the technical requirements of on-site multiphase flow measurement.
(2) Noise and adhesion-layer suppression. By jointly estimating the adhesion-layer thickness and the observation-noise covariance online, the algorithm effectively suppresses both the slowly-varying baseline drift caused by adhesion contamination and the high-frequency fluctuations induced by electromagnetic interference and temperature drift, thereby maintaining stable measurement under non-stationary field noise.
(3) Superiority over conventional filters. Under identical field conditions, all accuracy and error indicators of AUKF-PINN are significantly better than those of KF, EKF, UKF and the AUKF without PINN assistance, confirming that the physics-informed noise prior and the adaptive fusion strategy jointly provide the decisive performance gain rather than any single component in isolation.
(4) Engineering significance. The proposed method delivers reliable phase-fraction and mass-flow information for drilling-overflow early warning and for the optimization of managed-pressure-drilling control parameters, and provides an effective physics-enhanced filtering paradigm for multiphase flow measurement in complex industrial environments, showing broad engineering application prospects.

Future work will focus on extending the framework to a wider range of flow regimes and on validating its long-term stability in continuous field deployment.

## Supporting information

S1 File Data. (XLSX)
